# Swine acute diarrhoea syndrome coronavirus (SADS-CoV) Nsp5 antagonizes type I interferon signaling by cleaving DCP1A

**DOI:** 10.3389/fimmu.2023.1196031

**Published:** 2023-05-22

**Authors:** Hai-xin Huang, Chen-chen Zhao, Xiao-xiao Lei, Xin-yu Zhang, Yu-ying Li, Tian Lan, Bao-peng Zhao, Jing-yi Lu, Wen-chao Sun, Hui-jun Lu, Ning-yi Jin

**Affiliations:** ^1^ College of Veterinary Medicine, Northwest A&F University, Xianyang, China; ^2^ Institute of Virology, Wenzhou University, Wenzhou, China; ^3^ Changchun Institute of Veterinary Medicine, Chinese Academy of Agricultural Sciences, Changchun, China

**Keywords:** swine acute diarrhoea syndrome coronavirus (SADS-CoV), interferon signaling, mRNA-decapping enzyme 1a (DCP1A), nonstructural protein 5 (nsp5), antiviral activity

## Abstract

Swine acute diarrhoea syndrome coronavirus (SADS-CoV), which is a recently discovered enteric coronavirus, is the major aetiological agent that causes severe clinical diarrhoea and intestinal pathological damage in pigs, and it has caused significant economic losses to the swine industry. Nonstructural protein 5, also called 3C-like protease, cleaves viral polypeptides and host immune-related molecules to facilitate viral replication and immune evasion. Here, we demonstrated that SADS-CoV nsp5 significantly inhibits the Sendai virus (SEV)-induced production of IFN-β and inflammatory cytokines. SADS-CoV nsp5 targets and cleaves mRNA-decapping enzyme 1a (DCP1A) via its protease activity to inhibit the IRF3 and NF-κB signaling pathways in order to decrease IFN-β and inflammatory cytokine production. We found that the histidine 41 and cystine 144 residues of SADS-CoV nsp5 are critical for its cleavage activity. Additionally, a form of DCP1A with a mutation in the glutamine 343 residue is resistant to nsp5-mediated cleavage and has a stronger ability to inhibit SADS-CoV infection than wild-type DCP1A. In conclusion, our findings reveal that SADS-CoV nsp5 is an important interferon antagonist and enhance the understanding of immune evasion by alpha coronaviruses.

## Introduction

Swine acute diarrhoea syndrome coronavirus (SADS-CoV) is also known as swine enteric alphacoronavirus (SeACoV) or porcine enteric alpha coronavirus (PEAV), and it is a novel HKU2-related bat coronavirus that belongs to the alpha coronavirus family. SADS-CoV was first discovered during a catastrophic epidemic in pigs in Guangdong Province, China, in 2017, which led to the death of nearly 24,500 newborn piglets and caused significant economic losses (1–3). In 2018, seven pig farms in Fujian Province reported the presence of a new SADS-CoV strain in pig stool and small intestine samples (4). In 2019, the re-emergence of SADS-CoV infection in pig herds in southern China resulted in an outbreak of diarrhoea that caused approximately 2000 piglet deaths (5). An outbreak of a fatal swine diarrhoea disease occurred on an intensive-scale pig farm in Guangxi, China, in May 2021, and it led to more than 3000 deaths in piglets. The clinical symptoms of infected animals include severe acute diarrhoea, vomiting, and weight loss, and the mortality rate can reach 100% (6). SADS-CoV has been reported to infect a wide variety of cell lines in culture, including cell lines derived from its natural hosts (bats, monkeys, cats, dogs, minks, hamsters), susceptible hosts (pigs), and potential hosts (humans and other animals) (7, 8). Chickens are susceptible to infection with SADS-CoV under laboratory conditions, suggesting that chickens may be asymptomatic carriers of SADS-CoV and that the virus has the potential to spread to susceptible flocks near pig farms (9).

The full-length genome of SADS-CoV is approximately 27 kb in length, and it is arranged in the order of 5’UTR-ORF1a/1b-S-NS3-E-M-N-NS7a-NS7b-3’UTR. ORF1a and ORF1b encode polyprotein 1a (pp1a) and polyprotein 1b (pp1b), respectively, and pp1a and pp1b are processed into 16 nonstructural proteins (Nsp1-16) that are responsible for viral RNA synthesis. Maturation of viral proteins requires cleavage by proteases. Nonstructural protein 5 (nsp5), which is a 3 chymotrypsin-like protease (3CL^pro^, also called main protease), is an attractive drug target because it can recognize sequences containing Leu and Gln at positions P2 and P1 and play an important role in cleaving viral polyproteins to generate functional proteins (10, 11). To date, there are still no effective vaccination strategies or antiviral drugs to control SADS-CoV infection. The development of more effective control measures will be made easier with a better understanding of the mechanisms by which SADS-CoV evades the host immune system.

Interferons (IFNs) are a broad class of cytokines that are elicited upon challenge to host defences, and they are essential for mobilizing immune responses to pathogens and activating signal transduction cascades that lead to the induction of hundreds of interferon-stimulated genes (ISGs). As ISGs play important roles in antiviral responses, viruses develop strategies to counteract their functions (12). mRNA-decapping enzyme 1a (DCP1A), which is a regulatory factor that is involved in removing the 5’-methylguanosine cap from eukaryotic mRNAs, has recently been identified as an IFN-stimulated gene. The study suggested that DCP1A phosphorylation modulates the host chemokine response to respiratory syncytial virus (RSV) infection (13). The study reported that porcine reproductive and respiratory syndrome virus (PRRSV) infection significantly downregulates DCP1A expression at the protein level by cleaving DCP1A, and PRRSV nsp4 is responsible for DCP1A cleavage, specifically at glutamic acid 238 (E238) of DCP1A (14). Porcine delta coronavirus (PDCoV) nsp5 cleaves porcine DCP1A via its protease activity and cleaves DCP1A at glutamine 343 (Q343) (10). Severe acute respiratory syndrome coronavirus 2 (SARS-CoV-2) nsp5 inhibits HDAC-dependent ISG antiviral responses and abolishes the activity of the ISG effector DCP1A (15).

Currently, there are few reports about the pathogenesis of SADS-CoV, and there are no relevant reports about whether SADS-CoV nsp5 contributes to the escape of SADS-CoV from innate immune responses and whether it plays roles in SADS-CoV pathogenesis and transmission. And there is no effective vaccine or antiviral drug for SADS-CoV. The nsp5 of SADS-CoV provides an excellent target for antivirals, due to its essentialand conserved function in the viral replication cycle. In this study, we found that SADS-CoV nsp5 antagonized IFN-β production and targeted DCP1A, thus impairing the antiviral activity of DCP1A. Additionally, nsp5 from different CoVs cleaves DCP1A, suggesting a common mechanism underlying immune evasion. It may be possible to gain a better understanding of SADS-CoV disease pathogenesis and transmission by elucidating the mechanism underlying nsp5-mediated catalysis and IFN antagonism.

## Materials and methods

### Cell culture and virus

HEK-293T cells, LLC-PK1 cells, IPEC-J2 cells, Vero-E6 cells and ST cells were maintained in Dulbecco’s modified Eagle medium (DMEM) (HyClone, Logan, UT, USA) supplemented with 10% foetal bovine serum (FBS, Gibco, Grand Island, NY, USA) and penicillin–streptomycin at 37°C in a 5% CO_2_ atmosphere. The SADS-CoV strain was previously preserved in our laboratory. SADS-CoV was diluted in DMEM supplemented with 10 mg/mL trypsin and inoculated into Vero-E6 cells, which were replaced with fresh DMEM medium containing 2% FBS after 2 h. Vesicular stom atitis virus carrying the green fluorescent protein gene (VSV-GFP) and Sendai virus (SeV) were previously preserved in our laboratory.

### Plasmids, siRNA and transfection

Nsp5 from SADS-CoV was cloned into a pCAGGS-FLAG vector. Full-length cDNA sequences of *sus scrofa* (pig) DCP1A (GenBank accession number NM_001244358.1), *Homo sapiens* (human) DCP1A (GenBank accession number NM_001290204.2), and *Chlorocebus sabaeus* (green monkey) (GenBank accession number XM_007984575.2) were cloned into a pXJ40-HA vector. Full-length cDNA sequences of porcine IFIT1, IFIT3 and IFIT5 was also cloned into a pXJ40-HA vector. Vectors expressing P65, IKBKE, TRAF3, MDA-5, STAT1, STAT2, RIG-I, NEMO, IKKα, IKKβ, IκBα, IRF3 were purchased from HonorGene. *Homo sapiens* DCP1A small interfering RNAs (siRNAs) were designed and purchased from Tsingke Biotech (Beijing, China), together with the control siRNA. The constructs were transfected using Lipofectamine 3000 Reagent (Invitrogen) and siRNAs were transfected using Lipofectamine RNAi MAX (Invitrogen) according to procedures recommended by the manufacturer.

### Antibodies and reagents

A DYKDDDDK Tag(9A3) mouse mAb (#8146), HA-Tag (C29F4) rabbit mAb (#3724), phospho-NF-κB P65 (Ser536) rabbit mAb (#3033), phospho-IRF-3 (Ser386) rabbit mAb (#37829), Lamin A/C rabbit antibody (#2032), anti-mouse IgG(H+L), F(ab’)_2_ fragment (Alexa Fluor 488 Conjugate) (#4408), anti-rabbit IgG (H+L), and F(ab’)_2_ fragment (Alexa Fluor^®^ 594 Conjugate) (#8889) were purchased from Cell Signaling Technology (CST, Boston, MA, USA). A phospho-TBK1 (Ser172) rabbit antibody (#AF8190), phospho-IκBα (Ser32/Ser36) rabbit antibody (#AF2002), phospho-STAT1 (Tyr701) rabbit antibody (#AF3300), TBK1 rabbit antibody (#DF7026), phospho-STAT2 (Tyr690) antibody (#AF3342) were purchased from Affinity Biosciences (Cincinnati, OH, USA). A DCP1A rabbit mAb was purchased from ABclonal (#A6824) (Wuhan, China). A RSAD2 rabbit polyclonal antibody (#28089-1-AP), HRP-conjugated AffiniPure donkey anti-rabbit IgG (H+L) (#SA00001-9), IRF3 rabbit polyclonal antibody (#11312-1-AP), STAT1 rabbit polyclonal antibody (#10144-2-AP), STAT2 rabbit polyclonal antibody (#16674-1-AP), IKBKG rabbit polyclonal antibody (#18474-1-AP), NF-κB P65 rabbit polyclonal antibody (#10745-1-AP), His-Tag mouse monoclonal antibody (#66005-1-Ig), GAPDH monoclonal antibody (#60004-1-Ig), and HRP-conjugated AffiniPure goat anti-mouse IgG (H+L) were purchased from Proteintech Group (Rosemont, IL, USA). Z-VAD(OMe)-FMK (#T6013), PF-00835231 (#T9458), 3-MA (#T1879), and MG132 (#T2154) were purchased from Target Mol (Boston, USA). Poly(I:C) (#P1530) was purchased from Sigma−Aldrich (St. Louis, MO, USA), ProteinIso^®^ Protein A/G Resin (#DP501), and the TransDetect^®^ Double-Luciferase Reporter Assay Kit (#FR201) were purchased from TransGen Biotech (Beijing, China). The UNlQ-10 Column TRIzol Total RNA Isolation Kit was purchased from Sangon Biotech (#B511321) (Shanghai, China). PrimeScript™ RT Master Mix (Perfect Real Time) was purchased from TaKaRa (#RR037A) (Beijing, China). Taq Pro Universal SYBR qPCR Master Mix was purchased from Vazyme (#Q702) (Nanjing, China).

### Dual-luciferase reporter activity assays

When the cell confluence in 12-well plates reached approximately 80%, HEK-293T or ST cells were cotransfected with the reporter plasmid (IFN-β-Luc) at 0.2 μg/well and the plasmid pRL-TK (internal control for normalization of the transfection efficiency) at 0.01 μg/well with Lipofectamine 3000 reagent. After transfection for 24 h, the cells were lysed. Then, the levels of firefly luciferase and Renilla luciferase were measured using the Dual-Luciferase reporter assay system. The data are shown as the relative firefly luciferase activities normalized to the Renilla luciferase activities from three independent experiments.

### Confocal immunofluorescence assay

LLC-PK1 cells were transfected with porcine DCP1A, SADS-CoV nsp5 or nsp14 expression vectors. After 24 h, the cells were fixed with 4% paraformaldehyde for 20 min and then permeabilized with 0.1% Triton-X 100 in PBS. Then, the cells were incubated with 3% BSA in PBS for 2 h at room temperature. LLC-PK1 cells were then stained with the DYKDDDDK Tag(9A3) mouse mAb and DCP1A rabbit mAb diluted in 1% BSA in PBS at 4°C overnight. The cells were then incubated with fluorochrome-conjugated secondary Abs for 2 h at room temperature. After incubation with the secondary Abs, the specimens were covered with DAPI and incubated for 6 min to stain the nuclei. The images were collected by forwarding fluorescence microscopy and confocal microscopy.

### RNA isolation and quantitative PCR analysis

Total RNA was extracted from cultured cells using TRIzol reagent and reverse transcribed into cDNA using reverse transcriptase. Quantitative real-time PCR (RT−qPCR) experiments were performed in triplicate. Relative mRNA expression levels were normalized to the expression level of GAPDH. All the RT−qPCR experiments were performed using Taq Pro Universal SYBR qPCR Master Mix and an ABI QuantStudio 3 Real-time PCR system.

### Western blotting analysis

HEK-293T cells were transfected with the indicated plasmids as mentioned above, harvested after 30 h by the addition of lysis buffer, and incubated on ice for 15 min. The cell lysates were centrifuged at 12,000 × g for 15 min before the supernatants were either subjected to immunoprecipitation (IP) or directly denatured at 100°C for 10 min. The denatured cell lysates were separated by SDS−PAGE and transferred to PVDF membranes using a Trans-Blot^®^ SD Semi-Dry Electrophoretic Transfer cell at 15 V for 50 min. For immunoblotting, the indicated primary antibodies were incubated with the membranes for 2 h at room temperature or overnight at 4°C; HRP-conjugated goat anti-mouse or goat anti-rabbit IgG were used as secondary antibodies. The bands were visualized with chemiluminescent reagent (NCM Biotech, Shanghai, China) and were imaged by an ECL imaging system (Bio-Rad, Hercules, CA, USA). GAPDH expression in each sample was used as a control to demonstrate equal loading of the protein samples across lanes.

### Coimmunoprecipitation assay

HEK-293T cells were seeded in 10 cm dishes, transfected with the indicated plasmids as mentioned above and lysed at 30 h posttransfection in 1 mL lysis buffer per dish. The lysates were centrifuged at 14,000 × g for 15 min, and the supernatants were subjected to IP, with 100 μL reserved for use as a whole cell lysate (WCL) control. Briefly, ProteinIso^®^ Protein A/G Resin was added to 400 μL of cell lysate and incubated on a shaker at 4°C for 1 h. The mixture was centrifuged at 1,000 × g for 5 min, and the supernatant was extracted. Then, 2 μL of antibody was added to 400 μL of cell lysate and incubated on a shaker at 4°C overnight. Then, ProteinIso^®^ Protein A/G Resin (#DP501, TransGen Biotech, Beijing, China), which had been washed with lysis buffer three times, was added. The mixture was centrifuged at 1,000 × g for 5 min, and the sample was collected and washed in lysis buffer three times at 1,000 × g for 5 min. Next, the beads were mixed with 1× loading buffer, and the mixture was denatured at 100°C for 10 min before analysis along with the WCL by Western blotting.

### Statistical analysis

The data were analysed with GraphPad Prism 9 software, and Student’s t-test were performed to determine significance. The data are expressed as the mean ± standard deviation (SD) of three independent experiments. *P* values < 0.05 were considered statistically significant.

## Results

### SADS-CoV antagonizes type I IFN and inflammatory cytokine production

**Figure 1:**
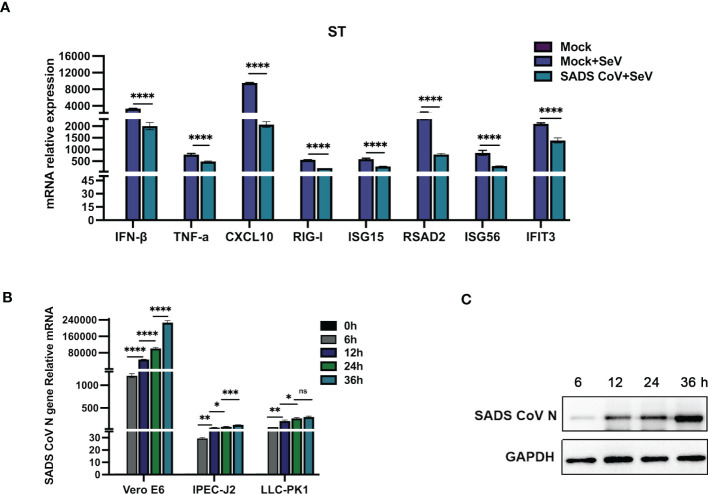
SADS-CoV antagonizes type I IFN and inflammatory cytokine production. **(A)** ST cells were cultured in 6-well plates infected with SADS-CoV. After 24 h, the cells were infected with 0.1 MOI SeV for an additional 12 h IFN-β, TNF-α, CXCL10, RIG-I, ISG15, RSAD2, ISG56, and IFIT3 mRNA levels were analysed by RT−qPCR. **(B)** Vero-E6, IPEC-J2, LLC-PK1 cells were infected with the SADS-CoV strain (MOI=0.1) and then harvested at 0 h, 6 h, 12 h, 24 h and 36 h to measure SADS-CoV N mRNA levels by RT−qPCR. **(C)** Vero-E6 cells (MOI=0.1) were infected with the SADS-CoV strain at 6 h, 12 h, 24 h and 36 h Cell lysates were analysed by Western blotting with anti-SADS-CoV N protein antibodies. All data are reported as mean±SD. For all experiments, *p < 0.05, **p < 0.01, and ***p < 0.001, ****p < 0.0001 were considered to be statistically significant. ns, nonsignificant differences in data.

Several coronaviruses have been reported to inhibit type I IFN production in order to evade host innate immunity (16–19). To investigate the effects of SADS-CoV infection on the expression of IFN-β and inflammatory cytokines in swine testicular (ST) cells, the mRNA expression levels of various factors were measured after viral infection. As shown in **Figure 1A**, compared with those in the control group, the SeV-induced mRNA expression levels of IFN-β, TNF-α, CXCL10, RIG-I, ISG15, RSAD2, ISG56, and IFIT3 were significantly inhibited by SADS-CoV. Next, we infected Vero-E6, IPEC-J2, and LLC-PK1 cells with SADS-CoV and analysed the proliferation of the virus and the copy number of the N gene by RT−qPCR (**Figure 1B**). We also measured the expression of the SADS-CoV N protein (**Figure 1C**). The results showed that the replication of viral genes and the expression of the N protein significantly increased with the time of infection.

### SADS-CoV nsp5 suppresses the type I IFN and NF-κB signaling pathways

**Figure 2:**
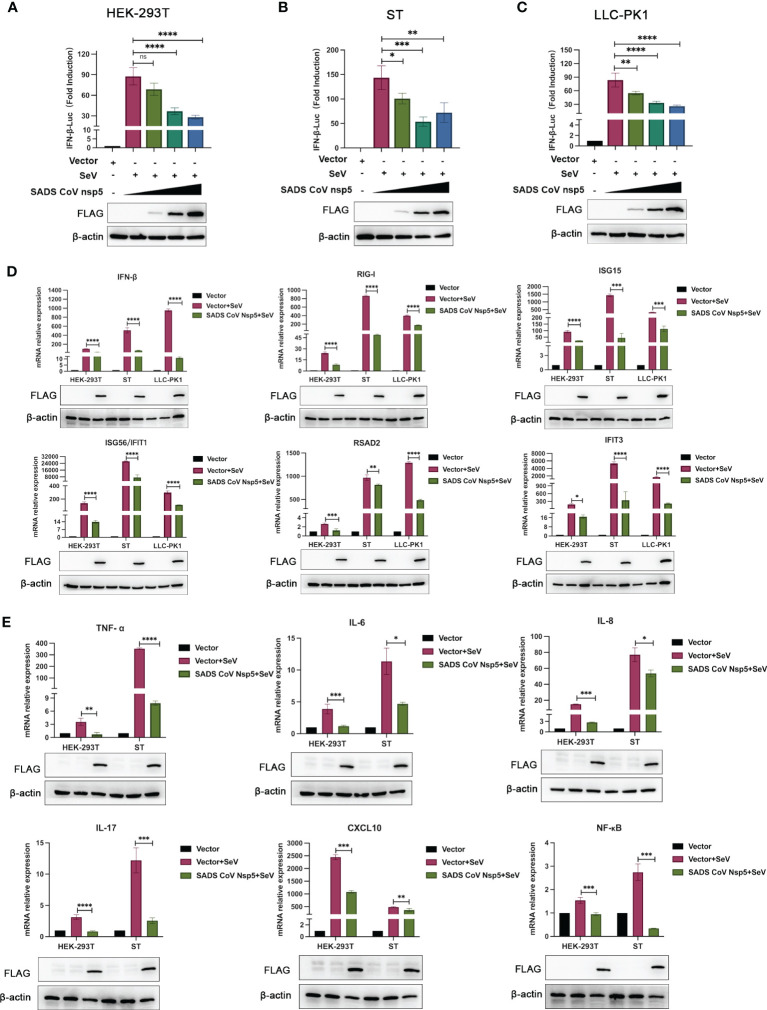
SADS-CoV nsp5 inhibits SeV-induced IFN-β and inflammatory cytokine production. **(A)** HEK-293T cells, **(B)** ST cells, or **(C)** LLC-PK1 cells were cotransfected with IFN-β Luc and pRL-TK plasmids, along with 1µg, 1.5 µg and 2.5µg of the SADS-CoV nsp5 plasmid for 24 h. Then, the cells were infected with or without SeV for 12 h. The cells were lysed and subjected to a dual-luciferase assay. And the expressions of SADS-CoV nsp5 protein and β-actin were detected by Western blot. **(D)** HEK-293T cells, ST cells and LLC-PK1 cells were transfected with 1.5 µg of the SADS-CoV nsp5 plasmid or an empty vector. After 24 h of initial transfection, the cells were further infected or mock infected with SEV. The cells and supernatants were collected at 12 h post infection, and the IFN-β, RIG-I, ISG15, RSAD2, ISG56, IFIT1, and IFIT3 mRNA levels were analysed by RT−qPCR. **(E)** HEK-293T cells and ST cells were transfected with 1.5 µg of the SADS-CoV nsp5 plasmid or an empty vector. After 24 h of initial transfection, the cells were further infected or mock infected with SEV. The cells and supernatants were collected at 12 h post infection, and the TNF-α, IL-6, IL-8, IL-17, CXCL10, and NF-κB mRNA levels were analysed by RT−qPCR. And the expressions of SADS-CoV nsp5 protein and β-actin were detected by Western blot. All data are reported as mean±SD. For all experiments, *p < 0.05, **p < 0.01, and ***p < 0.001, ****p < 0.0001 were considered to be statistically significant. ns, nonsignificant differences in data.

SeV infection strongly induced IFN-β promoter activity. However, transfection with nsp5 strongly inhibited the SeV-induced IFN-β promoter activity in HEK-293T, ST, and LLC-PK1 cells in a dose-dependent manner (**Figures 2A–C**). To assess the role of SADS-CoV nsp5 in regulating type I IFN signaling, we evaluated the mRNA levels of IFN-β, RIG-I, ISG15, RSAD2, ISG56, and IFIT3 after transfection of HEK-293T, ST, and LLC-PK1 cells with SADS-CoV nsp5. As shown in **Figure 2D**, compared with the control group, the transcription of ISGs induced by SeV was significantly inhibited by SADS-CoV nsp5. The role of nsp5 in regulating inflammatory cytokine signaling pathways was also evaluated. We evaluated the mRNA levels of TNF-α, IL-6, IL-8, IL-17, CXCL10, and NF-κB after transfection of HEK-293T and ST cells with nsp5 (**Figure 2E**). The results showed that nsp5 significantly suppressed the SeV-induced expression of inflammatory cytokines. To analyse the effect of SADS-CoV on the type I IFN and NF-κB signaling pathways, His-TBK1 and SADS-CoV nsp5 were cotransfected into HEK-293T cells, and the expression level of TBK1 was slightly decreased after SADS-CoV nsp5 expression. Transfection of nsp5 after poly(I:C) treatment reduced the level of phosphorylated IRF3 and inhibited NF-κB activation (**Supplementary Figure 1A**). In addition, the poly(I:C)-induced nuclear translocation of IRF3 was also limited. The protein levels of IRF3 in the cytoplasm of HEK-293T cells were increased by the expression of the nsp5 protein after poly(I:C) treatment. However, after poly(I:C) treatment, the IRF3 levels in the nucleus were decreased (**Supplementary Figure 1B**). As shown in **Supplementary Figures 1C, D**, transfection of nsp5 inhibited STAT1 expression and phosphorylation after poly(I:C) or IFN-β treatment as well as the phosphorylation of STAT2 and IκBα after poly(I:C) treatment.

### SADS-CoV nsp5 targets DCP1A for cleavage

**Figure 3:**
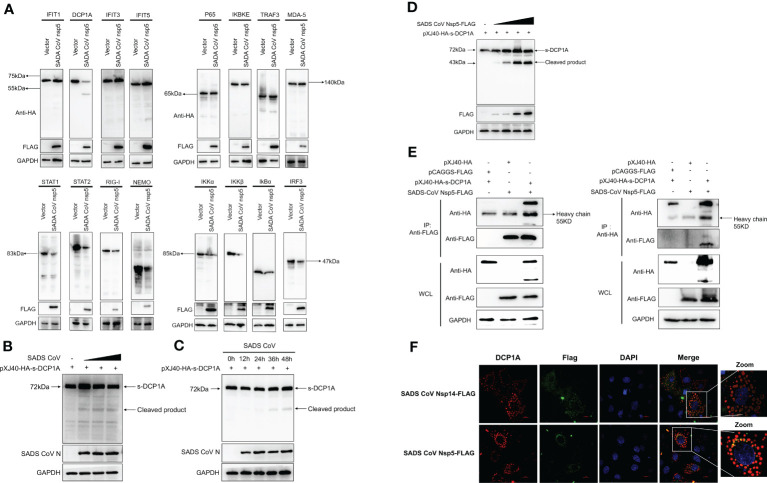
SADS-CoV nsp5 targets DCP1A for cleavage. **(A)** HEK-293T cells were cultured in 6-well plates and cotransfected with SADS-CoV nsp5 expression plasmid or empty vector along with 1.5 µg of HA-tagged IFIT1, DCP1A, IFIT3, IFIT5, P65, IKBKE, TRAF3, MDA-5, STAT1, STAT2, RIG-I, NEMO, IKKα, IKKβ, IκBα or IRF3 expression plasmid. After 28 h, the cells were lysed and analysed by Western blotting with an anti-HA antibody. **(B)** ST cells were transfected with 2 µg/well pXJ40-HA-sDCP1A expression plasmid. After 24 h of transfection, the ST cells were infected with SADS-CoV at different MOIs. **(C)** ST cells were infected with SADS-CoV strain (MOI=0.1) and then harvested at 0h, 12h, 24h, 36h, 48h, the cells were lysed for Western blotting. **(D)** HEK-293T cells were cotransfected with pXJ40-HA-sDCP1A and various amounts of the SADS-CoV nsp5 expression plasmid. After 30 h, the cells were lysed for Western blotting. **(E)** HEK-293T cells were transfected with expression constructs encoding SADS-CoV nsp5 and pXJ40-HA-sDCP1A. The cells were lysed 30 h after transfection and subjected to immunoprecipitation with an anti-FLAG antibody or anti-HA antibody. The whole-cell lysates (WCL) and immunoprecipitation (IP) complexes were analysed by immunoblotting (IB) using anti-FLAG, anti-HA, or anti-GAPDH antibodies. **(F)** LLC-PK1 cells were transfected with SADS-CoV nsp5 and nsp14. At 24 h, cells were fixed and then stained with an anti-rabbit monoclonal antibody against DCP1A and anti-mouse flag Tag antibody, followed by incubation with an Alexa Fluor 488-conjugated goat anti-mouse IgG antibody (green) or 594-conjugated goat anti-rabbit IgG antibody (red). The nuclei were stained with DAPI (blue).

We hypothesized that SADS-CoV nsp5 inhibits IFN-β and NF-κB production by inhibiting their upstream stimulatory molecules. Therefore, we cotransfected SADS-CoV nsp5 and pXJ40-HA- IFIT1, DCP1A, IFIT3, IFIT5, P65, IKBKE, TRAF3, MDA-5, STAT1, STAT2, RIG-I, NEMO, IKKα, IKKβ, IκBα or IRF3 into HEK-293T cells. The results showed that the expression levels of DCP1A, STAT1, STAT2, RIG-I, NEMO, IKKα, IKKβ, IκBα and IRF3 were decreased when SADS-CoV nsp5 was expressed, and the effect on DCP1A was the most significant (**Figure 3A**). Additionally, we also verified the effect of SADS-CoV on the expression of DCP1A, and we found that SADS-CoV infection significantly reduced the expression of DCP1A in a dose-dependent manner (**Figure 3B**). In addition, we examined the cleavage effect of SADS-CoV on DCP1A at different time points of infection in ST cells (**Figure 3C**). Further studies on the regulatory effect of SADS-CoV nsp5 on DCP1A expression showed that nsp5 could cleave DCP1A and produce obvious cleavage products; the levels of these cleavage products depended on the dose of SADS-CoV nsp5 (**Figure 3D**). Next, we verified the interaction between nsp5 and DCP1A by a coimmunoprecipitation assay. Flag-nsp5 and HA-DCP1A were cotransfected into HEK-293T cells, and immunoprecipitation (IP) experiments using anti-HA or anti-Flag antibodies showed that SADS-CoV nsp5 did indeed interact with DCP1A (**Figure 3E**). The interaction between SADS-CoV nsp5 and endogenous DCP1A was further verified by IFA colocalization experiments. In LLC-PK1 cells, the fluorescence signal of Flag-nsp5 overlapped with that of DCP1A. However, the fluorescence signal of Flag-nsp14 did not overlap with that of DCP1A (**Figure 3F**).

### DCP1A activates the type I IFN signaling pathway

**Figure 4:**
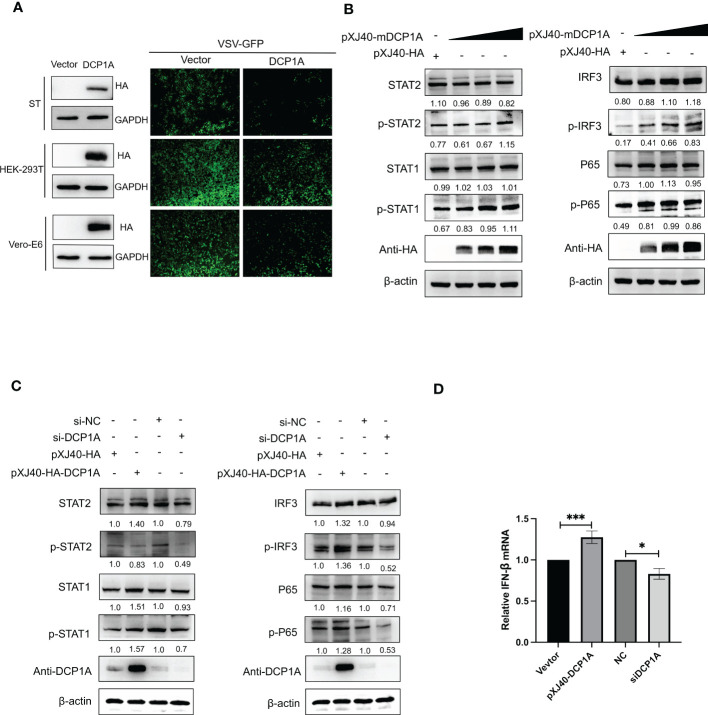
DCP1A activates the type I IFN signaling pathway. **(A)** ST, HEK-293T, and Vero-E6 cells were transfected with 2 µg/well empty vector or pXJ40-HA-sDCP1A, pXJ40-HA-hDCP1A, and pXJ40-HA-mDCP1A expression plasmids. After 24 h of transfection, the cells were infected with VSV-GFP for 12 h, and the expression of DCP1A was examined by Western blotting with an anti-HA antibody. Then, the replication of VSV-GFP was analysed via fluorescence microscopy. **(B)** HEK-293T cells were transfected with pXJ40-HA vector and various amounts of the pXJ40-HA-mDCP1A expression plasmid. **(C)** pXJ40-HA vector, pXJ40-HA-h-DCP1A expression plasmid, DCP1A siRNA and negative control siRNA were transfected into HEK-293T cells. After 30 h, cells were collected for Western blot analysis. Cells were analyzed by Western blotting with anti-STAT2, anti-phospho STAT2, anti-STAT1, anti-phospho STAT1, anti-IRF3, anti-phospho IRF3, anti-P65, anti-phospho P65, anti-HA, and β-actin antibodies. **(D)** pXJ40-HA vector, pXJ40-HA-h-DCP1A expression plasmid, DCP1A siRNA and negative control siRNA were transfected into HEK-293T cells. After 30 h, IFN-β mRNA levels were analysed by RT−qPCR. All data are reported as mean±SD. For all experiments, *p < 0.05 and ***p < 0.001 were considered to be statistically significant.

It has been reported that overexpression of DCP1A causes decreases in the RSV-induced expression of IL-8, indicating its ability to regulate cellular chemokine expression (13). Therefore, we hypothesized that DCP1A may be the upstream molecule that regulates IFN-β and NF-κB. We then transfected HEK-293T, ST, and Vero-E6 cells with pXJ40-HA-DCP1A and infected them with VSV-GFP. As shown in **Figure 4A**, the replication of VSV-GFP was significantly inhibited in DCP1A-expressing cells, suggesting that overexpression of DCP1A inhibited viral infection. We then overexpressed DCP1A to various degrees in HEK-293T cells and measured the expression of IRF3, STAT, and NF-κB. The results showed that the phosphorylation of STAT1, STAT2, IRF3, and P65 was markedly increased with increasing DCP1A expression (**Figure 4B**). Next, we used siRNA to knock down the expression of DCP1A and overexpress DCP1A with pXJ40-HA-DCP1A. The results showed that reducing the expression of DCP1A inhibited the activation of STAT1, STAT2, IRF3, and P65, and conversely increasing the expression of DCP1A promoted their expression (**Figure 4C, D**). These results indicate that DCP1A is a key regulator of the IFN signaling pathway.

### SADS-CoV nsp5-mediated of DCP1A depends on its protease activity

**Figure 5:**
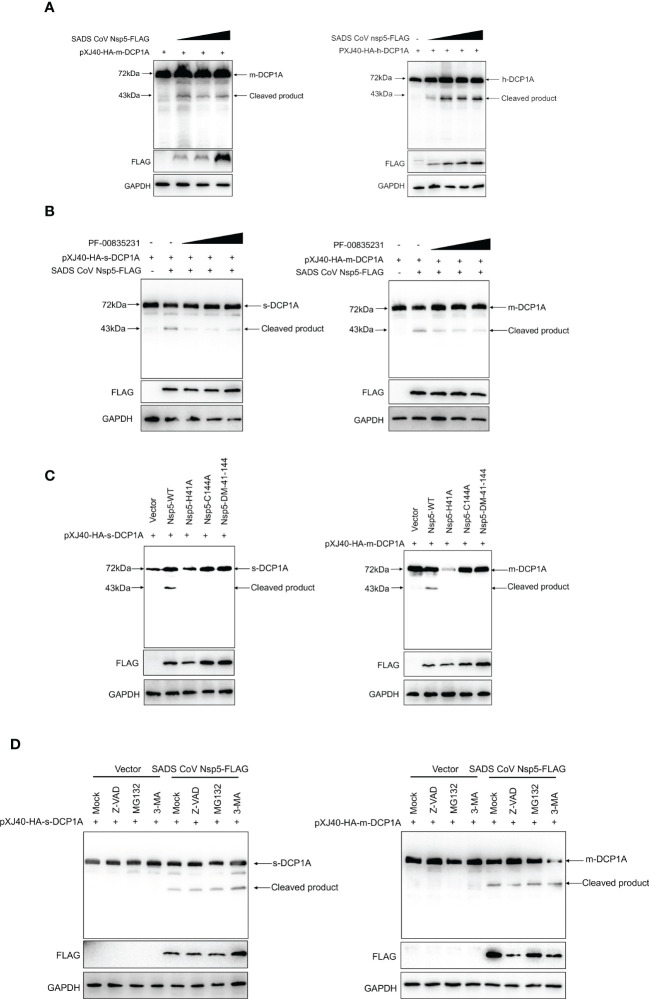
SADS-CoV nsp5 cleaves DCP1A via its protease activity. **(A)** HEK-293T cells were cotransfected with pXJ40-HA-mDCP1A, pXJ40-HA-hDCP1A and various amounts of the SADS-CoV nsp5 expression plasmid. After 30 h, the cells were lysed for Western blotting. **(B)** HEK-293T cells were cotransfected with plasmids encoding sDCP1A or mDCP1A and nsp5 from SADS-CoV. At 24 h after transfection, the cells were mock treated or treated with PF-00835231. After 12 h, the cells were lysed for Western blotting. **(C)** HEK-293T cells were cotransfected with plasmids expressing the wild-type SADS-CoV nsp5 or its protease-defective mutants (C144A and H41A) and the pXJ40-HA-sDCP1A or pXJ40-HA-mDCP1A expression plasmid. **(D)** HEK-293T cells were cotransfected with plasmids expressing the wild-type SADS-CoV nsp5 and pXJ40-HA-sDCP1A or pXJ40-HA-mDCP1A expression plasmids. After 24 h, the cells were treated with MG132, Z-VAD-FMK (final concentration of 20 µM) or 3-MA for 8 h Cell lysates were prepared and analysed by Western blotting.

Nsp5 proteins from several coronavirus-related viruses have also been shown to cleave host proteins and disrupt molecular pathways that are involved in innate immunity (20–22). To further determine whether SADS-CoV nsp5 cleaves DCP1A proteins from different species, we constructed pXJ40-HA-mDCP1A (monkey) derived from Vero-E6 cells, pXJ40-HA-sDCP1A (swine) derived from LLC-PK1 cells and pXJ40-HA-hDCP1A (human) derived from HEK-293T cells. Then, we cotransfected HEK-293T cells with DCP1A from different species and SADS-CoV nsp5. The results showed that the cleavage products of DCP1A increased with increasing SADS-CoV nsp5 expression (**Figure 5A**), and treatment with the coronavirus nsp5 inhibitor PF-00835231 significantly reduced the cleavage of sDCP1A and mDCP1A by SADS-CoV nsp5 (**Figure 5B**). To further investigate whether SADS-CoV nsp5-mediated cleavage of DCP1A depends on its protease activity, we constructed two mutants of nsp5 (H41A and C144A) with mutations that targeted its protease active sites (10), and we cotransfected these mutants with pXJ40-HA-mDCP1A and pXJ40-HA-sDCP1A, respectively. As shown in **Figure 5C**, the two nsp5 mutants H41A and C144A were unable to cleave mDCP1A and sDCP1A. Then, HEK-293T cells were cotransfected with SADS-CoV nsp5 and DCP1A and then treated with the proteasome inhibitor MG132, the caspase inhibitor Z-VAD-FMK, or the autophagy inhibitor 3-MA. The results showed that none of the three inhibitors could block the cleavage of DCP1A by SADS-CoV nsp5, indicating that the cleavage of DCP1A by nsp5 was dependent on the protease activity of nsp5 (**Figure 5D**).

### SADS-CoV nsp5 recognizes and cleaves DCP1A at residue Q343

**Figure 6:**
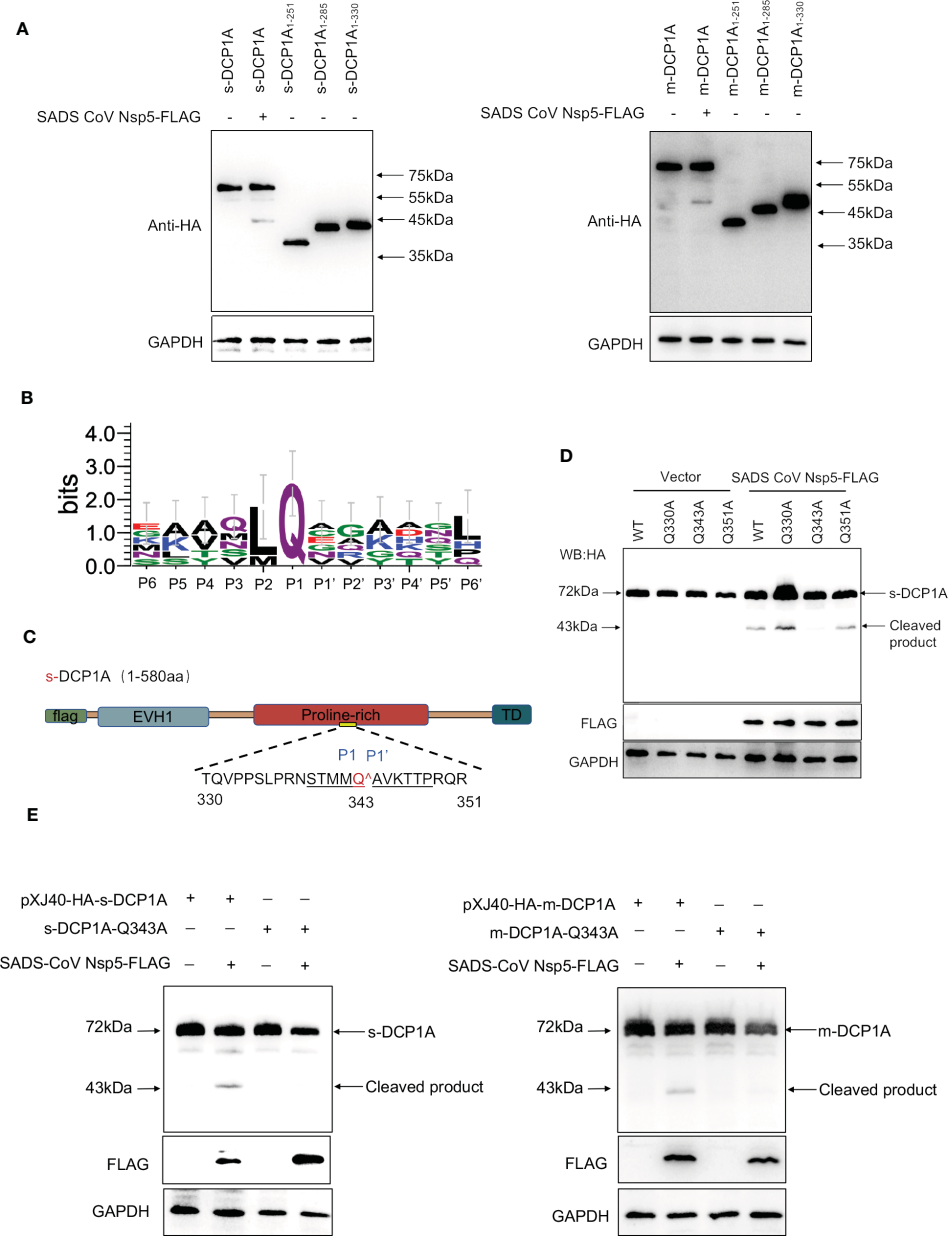
SADS-CoV nsp5 mediates DCP1A cleavage at residue Q343. **(A)** The estimated product of DCP1A cleavage by SADS-CoV nsp5. HEK-293T cells were transfected with expression constructs encoding mDCP1A_1–251_ or sDCP1A_1-251_, mDCP1A_1–285_ or sDCP1A_1–285_, mDCP1A_1–330_ or sDCP1A_1-330_ truncated mutants and collected after 30 h for Western blotting. **(B)** Sequence logo of the polyprotein junctions that are cleaved by SADS-CoV nsp5. An amino acid sequence logo of the substrate was generated by WebLogo, version 3 (http://weblogo.threeplusone.com/). **(C)** Schematic representation of mDCP1A and its mutant sites. **(D)** HEK-293T cells were cotransfected with the SADS-CoV nsp5 expression plasmid along with expression constructs encoding wild-type mDCP1A or mDCP1A mutants, including mDCP1A-Q330A, mDCP1A-Q343A, and mDCP1A-Q351A. The cells were then lysed after 30 h and evaluated by Western blotting. **(E)** HEK-293T cells were cotransfected with the SADS-CoV nsp5 expression plasmid along with wild-type sDCP1A/mDCP1A or sDCP1A-Q343A/mDCP1A-Q343A and collected after 30 h for Western blotting.

To further identify the site of DCP1A that is recognized by SADS-CoV nsp5, we generated three DCP1A truncation mutants, namely, DCP1A_1–251_, DCP1A_1–285_ and DCP1A_1–330,_ from different species, and we cotransfected them with nsp5. Since the size of the product that is recognized by anti-HA antibodies after the nsp5-mediated cleavage of sDCP1A and mDCP1A were approximately 43 kDa in size, the cleavage site should be near the 330 amino acid position in the N-terminus (**Figure 6A**). As shown in **Figure 6B**, CoV nsp5 showed a preference for substrate cleavage at a Q residue in the P1 position, which is common among other CoV subfamilies. Since the protease activity site of nsp5 usually recognizes Q-Gln motifs (23), we constructed several DCP1A constructs with mutations between amino acids 285 and 360 in the N-terminus, where Q residues (Q330, Q343, and Q351) were mutated to alanine residues, and we cotransfected these constructs with SADS-CoV nsp5 into HEK-293T cells (**Figure 6C**). The results showed that the DCP1A-Q330A and DCP1A-Q351A mutants from different species could still be cleaved by SADS-CoV nsp5, while the DCP1A-Q343A mutants were resistant. These results indicate that amino acid Q343 of DCP1A is a recognition and cleavage site for nsp5 (**Figures 6D, E**).

### DCP1A is a common target of nsp5 from different coronaviruses

Coronavirus nsp5 has a catalytic site between cysteine (Cys) residues and histidine (His) residues, and any change in the catalytic site disrupts its protease activity (24–26). Comparison of the secondary structure of nsp5 showed that the His41 and Cys144 residues of the protease active site (numbering based on SADS-CoV nsp5) were highly conserved among α-, β-, γ-, and δ-CoV (**Supplementary Figure 2A**). Therefore, we investigated whether nsp5 from other mammalian coronaviruses can also cleave DCP1A in the host. The results showed that nsp5 from different coronaviruses could cleave DCP1A in infected cells (**Supplementary Figures 2B, C**). These results demonstrate that DCP1A cleavage is a conserved mechanism by which coronavirus escapes host immune responses.

## Discussion

Here, we found that DCP1A is a possible target of SADS-CoV during infection. We show that following host infection, SADS-CoV nsp5 uses its protease activity to cleave DCP1A at residue Q343, and nsp5 from many different coronavirus species can also cleave DCP1A. The results reveal a previously unknown mechanism of intracellular antagonism that is driven by virus−host interactions, and these results provide new evidence to explain why hosts often exhibit limited ISG responses during SADS-CoV infection.

SADS-CoV was infectious in pigs when inoculated orally into 3-day-old newborn piglets, leading to clinical signs of diarrhea and subclinicalinfection (3). SADS-CoV has been reported to have a broad cell tropism, Vero-E6, IPEC-J2, ST and LLC-PK1 have been showed signicant susceptibility to SADS-CoV infection (27). In the immune system, IFN/ISG plays a crucial role in preventing virus infection in cells. We demonstrated that SADS-CoV inhibits the secretion of type I IFN and multiple inflammatory cytokines in ST cells. In our study, we also confirmed that SADS-CoV infected in different host cells (**Figure 1**). NF-κB is the primary transcription factor that regulates numerous cellular responses, including early innate immune responses and viral infections (28). It has also been reported that SARS-CoV-2 nsp5 enhances cytokine production by activating the NF-κB signaling pathway (29). In this study, we found that SADS-CoV nsp5 antagonizes the host antiviral response by inhibiting the IRF3, STAT, and NF-κB signaling pathways, reducing the secretion of antiviral cytokines by cells and thereby allowing the virus to evade host innate immunity (**Figure 2** and **Supplementary Figure 1**).

Coronavirus nsp5 is a cysteine protease that is required to process viral polyproteins, so it is essential for viral replication and can antagonize host immune proteins, promoting viral escape from host immune responses and replication. Nsp5 proteins from several coronaviruses have been reported to cleave host proteins, disrupting molecular pathways that are involved in innate immunity. For example, nsp5 proteins PEDV and PDCoV target NEMO and cleave it at a unique site (Q231), inhibiting the host innate immune response and promoting viral proliferation (30, 31). PEDV nsp5 inhibits pyroptosis by cleaving GSDMD, which is a key executioner of pyroptosis, at amino acids R238, T239, and F240 (22). SARS-CoV-2 nsp5 prevents the autophagic degradation of viral membrane proteins by cleaving the selective autophagy receptor SQSTM1/p62 (21). In addition, histone deacetylases (HDACs) have a significant effect in inhibiting viral infection. SARS-CoV-2 and PDCoV nsp5 can recognize and cleave HDACs to antagonize their antiviral effects and promote their own proliferation (15, 32). In this study, we demonstrated that the nsp5 protein from SADS-CoV directly interacts with DCP1A and cleaves DCP1A to disrupt the innate immune pathways of the host and escape from the host immune response (**Figure 3**). We also found that DCP1A is a key molecule in the regulation of the IRF3, STAT and NF-κB pathways, induces the secretion of downstream type I IFN and inflammatory cytokines, and is an important molecule for host antiviral innate immunity (**Figure 4**). Since cysteine (Cys) and histidine (His) are the major sites of coronavirus nsp5 protease activity, mutations in these catalytic sites disrupts nsp5 activity (33). Coronavirus nsp5 proteins target DCP1A at a unique site (Q343) to disrupt its antiviral activity, revealing a common recognition mechanism that is used to suppress the host innate immune response (10). In this study, we used mutant with mutations in the protease active site, namely, H41A and C144A, to confirm the protease active site of SADS-CoV nsp5. Additionally, a truncated form of DCP1A was used to confirm that the Q343 site is the site that is recognized and cleaved by SADS-CoV nsp5 (**Figures 5**, **6**).

**Figure 7:**
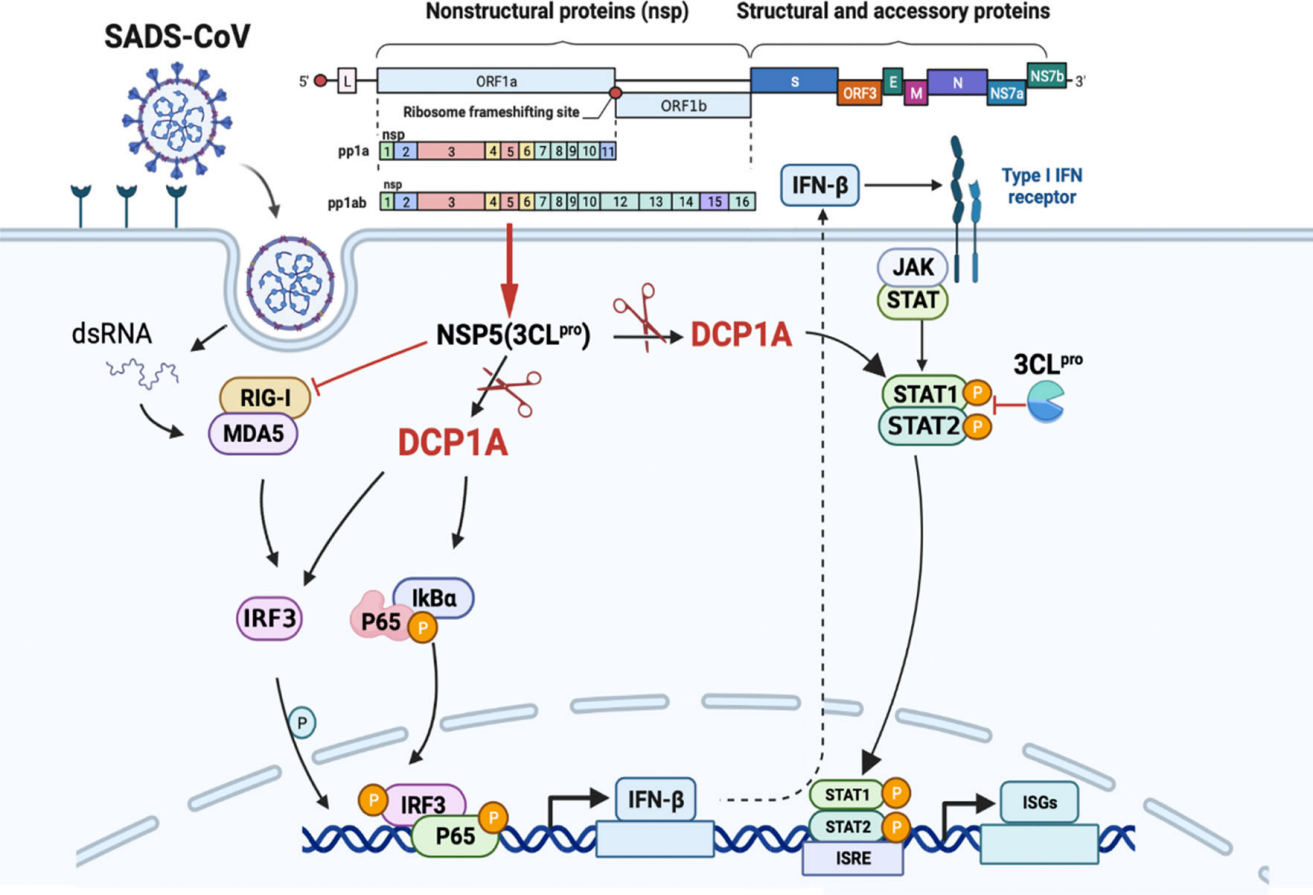
SADS-CoV nsp5 interferes with the innate immune response by directly cleaving DCP1A. SADS-CoV nsp5 antagonizes the type I interferon signaling pathway; the schematic was drawn using the Biorender website (https://www.biorender.com). SADS-CoV infects swine cells and releases its genome into the cytoplasm to produce nsp5 protein, which binds to DCP1A and cleaves it, inhibiting the activation of downstream molecules IRF3, NF-κB, STAT1, and STAT2, thereby reducing the production of type I IFN and inflammatory cytokines in the cells.

This specific and identifiable mode of cleavage via protease activity that targets a constant site is an effective way for coronaviruses to combat host innate immunity. It has been reported that DCP1A is a good antiviral target due its interaction with the nsp5 proteins from SARS-CoV-2 and PDCoV. We also verified that the nsp5 proteins from alpha-, beta- and delta-coronaviruses can target DCP1A for cleavage (**Supplementary Figure 2**). Therefore, further detailed analysis of the complete crystal structure of the viral nsp5 protein and elucidation of the molecular mechanism underlying its target gene recognition will provide more guidance for designing anti-coronavirus drugs that specifically targeting nsp5. According to the schematic diagram showing the molecular mechanism, these findings advance our understanding of the role of nsp5 during SADS-CoV infection (**Figure 7**).

In conclusion, these data contribute to a better understanding of the potential mechanisms by which SADS-CoV fights host innate immunity, increases its pathogenicity and infectivity in the host, and promotes its own replication and transmission. This study provides new insights for the prevention and control of SADS-CoV in clinical practice and provides guidance for the research and development of anti-SADS-CoV drugs and inhibitors in the future.

## Data availability statement

The original contributions presented in the study are included in the article/**Supplementary Material**. Further inquiries can be directed to the corresponding authors.

## Author contributions

N-YJ and W-CS designed the research. H-XH and C-CZ performed the majority of the experiments and were involved in preparation of the manuscript. H-XH, C-CZ, X-XL, X-YZ, and Y-YL analysed the data. H-XH prepared the manuscript. TL, B-PZ, J-YL, and H-JL revised the manuscript. All authors contributed to the article and approved the submitted version.
