# Supplementary material for: Swine acute diarrhoea syndrome coronavirus (SADS-CoV) Nsp5 antagonizes type I interferon signaling by cleaving DCP1A
Source: Front Immunol. 2023 May 22;14:1196031. doi: 10.3389/fimmu.2023.1196031 (PMC10239798; doi:10.3389/fimmu.2023.1196031)
Supplement: Supplementary file 1 [file DataSheet_1.pdf]

Supplementary Material

Swine acute diarrhoea syndrome coronavirus (SADS-CoV) Nsp5 antagonizes type I interferon signaling by cleaving DCP1A

Hai-xin Huang[1,2†], Chen-chen Zhao[2†], Xiao-xiao Lei[2], Xin-yu Zhang[2], Yu-ying Li[2], Tian Lan[2], Bao-peng Zhao[2], Jing-yi Lu[2] , Wen-chao Sun[2*], Hui-jun Lu[3*] and Ning-yi Jin[1,3*]

[1]*College of Veterinary Medicine, Northwest A&F University, Xianyang, China,*

[2]*Institute of Virology, Wenzhou University, Wenzhou, China,*

[3]*Changchun Institute of Veterinary Medicine, Chinese Academy of Agricultural Sciences, Changchun, China.*

*** Correspondence:**

Corresponding author

Ning-yi Jin, ningyik@126.com, Hui-jun Lu, huijun_lu@126.com, Wen-chao Sun, sunwenchao131@163.com

Supplementary Figures and Tables

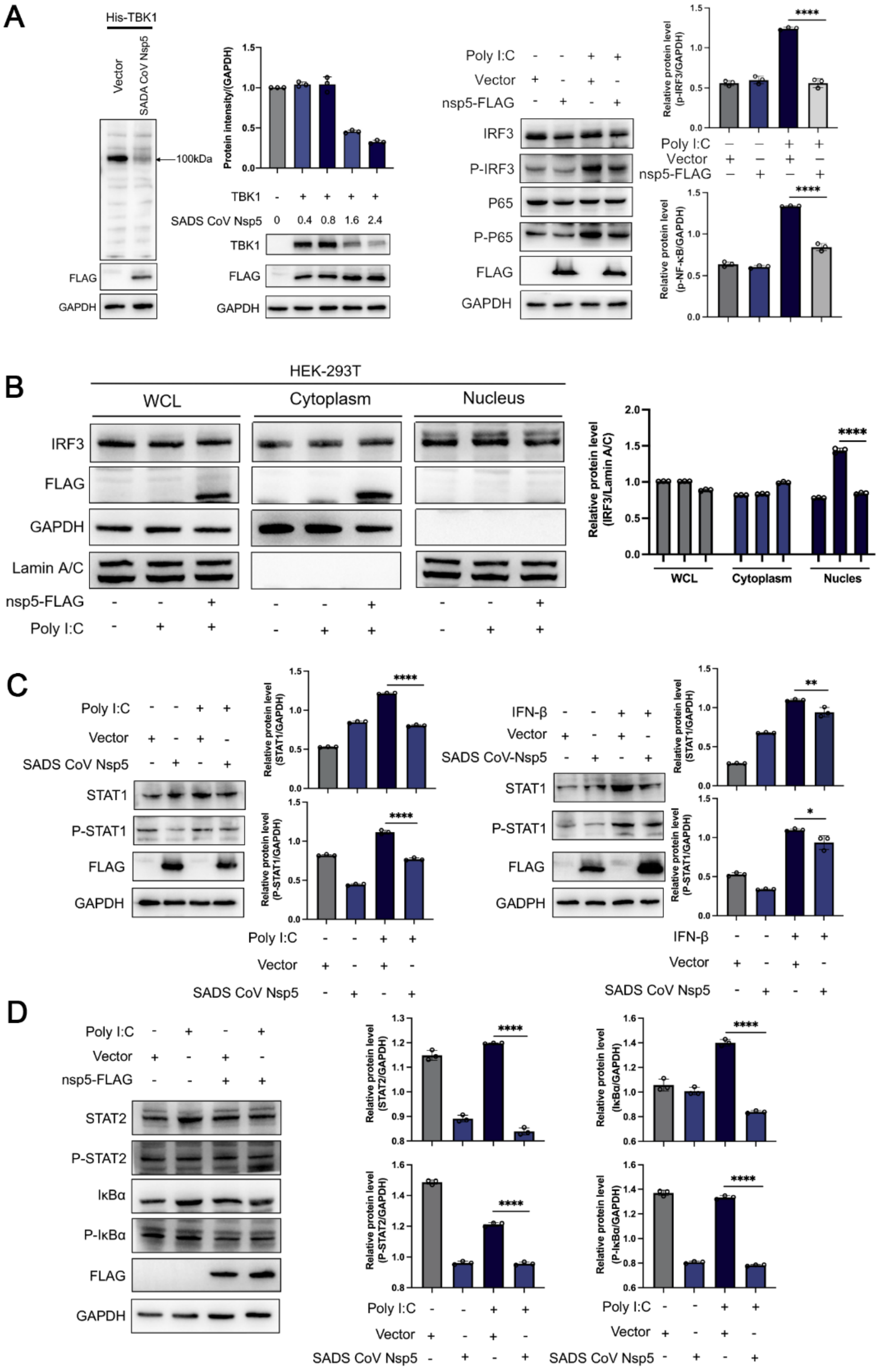

Supplementary figure 1. SADS-CoV nsp5 antagonizes the type I IFN signaling pathway. (A) HEK-293T cells were cotransfected with His-TBK1 and the SADS-CoV nsp5 expression plasmid. HEK-293T cells were cotransfected with His-TBK1 and various amounts of the SADS-CoV nsp5 expression plasmid. After 30 h, the cells were lysed for Western blotting. Relative expression ratios of TBK1 were quantified and normalized to GAPDH expression using Image J. HEK-293T cells were transfected with the indicated SADS-CoV nsp5 expression plasmid. After 24 h of transfection,

the cells were stimulated with poly(I:C) (2 μg/mL) for 12 h. Cell lysates were analysed by Western blotting with anti-IRF3, anti-phospho-IRF3, anti-P65, anti-phospho-P65, anti-FLAG and anti-GAPDH antibodies. Relative expression ratios of phospho-IRF3 and phospho-P65 were quantified and normalized to GAPDH expression using Image J. (B) HEK-293T cells were transfected with the SADS-CoV nsp5 expression plasmid for 24 h and treated with poly(I:C) (2 μg/mL) for 12 h. IRF3 levels in the nuclear and cytoplasmic fractions were determined by immunoblotting analyses. GAPDH served as a cytoplasmic control, and Lamin A/C served as a nuclear protein control. (C) HEK-293T cells were transfected with the SADS-CoV nsp5 expression plasmid for 24 h, stimulated with poly(I:C) for 12 h and treated with IFN-β for 8 h. Cell lysates were analysed by Western blotting with anti-STAT1, anti-phospho-STAT1, anti-FLAG and anti-GAPDH antibodies. Relative expression ratios of phospho-STAT1 were quantified and normalized to GAPDH expression using Image J. (D) HEK-293T cells were transfected with the indicated SADS-CoV nsp5 plasmid. After 24 h of transfection, the cells were stimulated with poly(I:C) for 12 h. Cell lysates were analysed by Western blotting with anti-STAT2, anti-phospho-STAT2, anti-IκBα, anti-phospho-IκBα, anti-FLAG and anti-GAPDH antibodies. Relative expression ratios of phospho-STAT2 and phospho-IκBα were quantified and normalized to GAPDH expression using Image J.

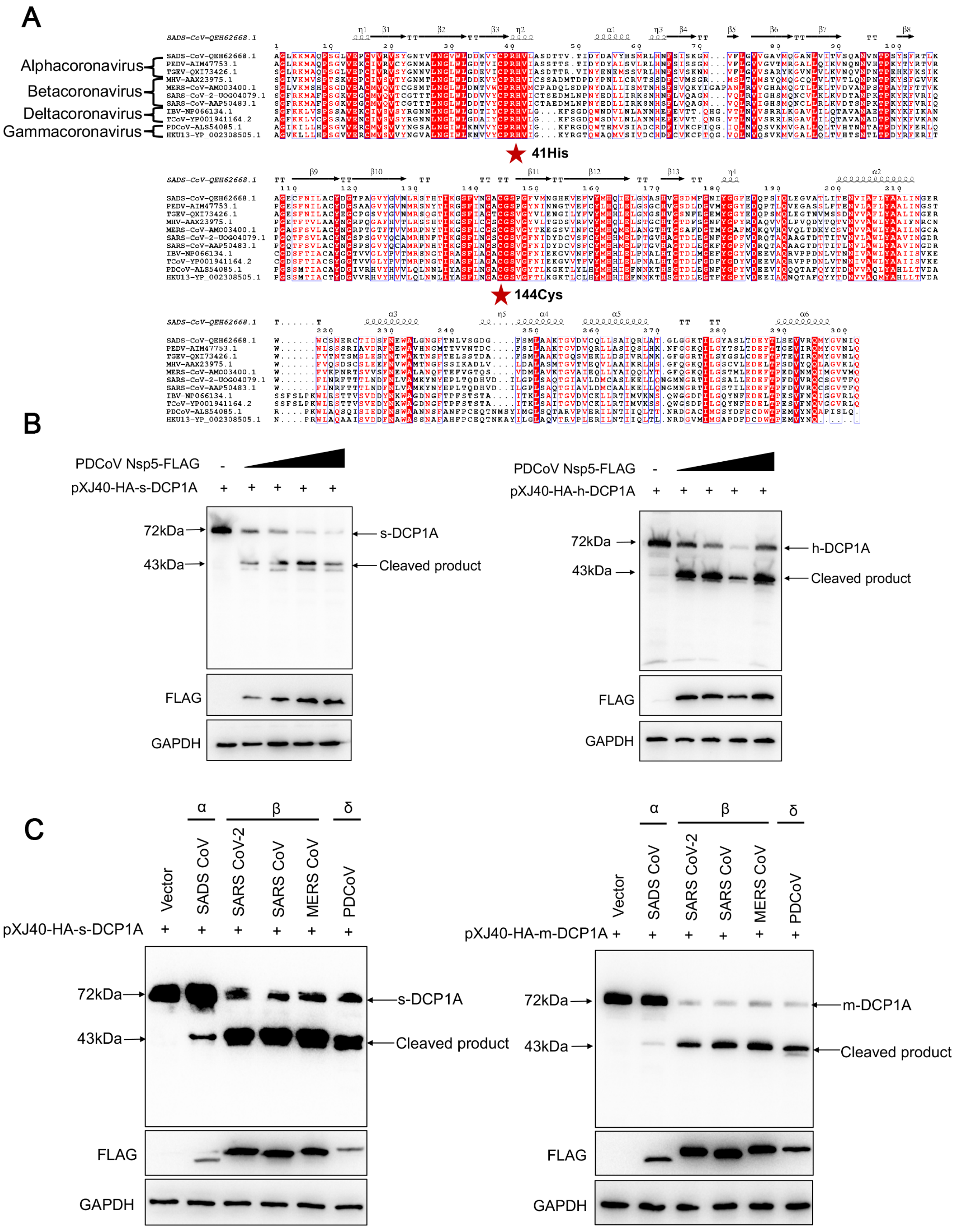

Supplementary figure 2. DCP1A is a common target of nsp5 from different coronaviruses. (A) Sequence alignment of nsp5 from the four genera of CoVs. Alpha-CoVs were represented by SADS-CoV (GenBank accession number QEH62668.1) and PEDV (GenBank accession number AIM47753.1) and TGEV (GenBank accession number QXI73426.1); Beta-CoVs were represented by MERS CoV (GenBank accession number AM003400.1), MHV (GenBank accession number AAX23975.1), SARS-CoV-2 (GenBank accession number UOG04079.1) and SARS CoV (GenBank accession number AAP50483.1); Gamma-CoVs were represented by IBV (GenBank

accession number NP066134.1) and TCoV (GenBank accession number YP001941164.2); and Delta-CoVs were represented by PDCoV (GenBank accession number ALS54085.1) and HKU13 (GenBank accession number YP_002308505.1). Secondary structures of CoV nsp5 proteins were analysed using the ESPript website (http://espript.ibcp.fr/ESPript/ESPript/index.php). Secondary structural elements of SADS-CoV nsp5 proteins are represented as spirals for α-helix, η for 310 helix, arrows for β-strands, and T for β-turns. Residues that are conserved among all CoV nsp5 proteins are represented in white on a red background. Residues that are conserved among most CoV nsp5 proteins are presented in red and boxed with a white background. (B) HEK-293T cells were cotransfected with pXJ40-HA-sDCP1A, pXJ40-HA-hDCP1A and various amounts of PDCoV nsp5. After 30 h, the cells were lysed for Western blotting. (C) HEK-293T cells were cultured in 6-well plates and cotransfected with mDCP1A or sDCP1A expression plasmid and nsp5 protein from SADS-CoV, SARS-CoV-2, SARS-CoV, MERS-CoV or PDCoV. After 30 h, the cells were lysed and analysed by Western blotting.

Table 1. Real-time PCR primers used in this study.

Primer	Forward (5'-3')	Reverse (5'-3')
p-IFN-β	CATCCTCCAAATCGCTCTCC	ACATGCCAAATTGCTGCTCC
p-TNF-α	CCAGACCAAGGTCAACCTCC	TCCCAGGTAGATGGGTTCGT
p-IL-6	AGGGAAATGTCGAGGCTGTG	TCCACTCGTTCTGTGACTGC
p-IL-8	AGAGTGGACCCCACTGTGAA	TGTACAACCTTCTGCACCCA
p-CXCL10	ACTGTTCGCTGTACCTGCAT	GCTTCTCTCTGTGTTCGAGGA
p-RIG-I	CTGGAGCTTGCTTTACCTGC	CCTTCCCCTTTCGTCCTTGT
p-ISG-15	TTGAGGGACTGCATGATGGC	CCAGGATGCTCAGTGGGTCT
p-NF-κB	CCTGAGGCTATAACTCGCTTGG	GTCCGCAATGGAGGAGAAGT
p-ISG-56	TCCGACACGCAGTCAAGTTT	TGTAGCAAAGCCCTGTCTGG
p-IFIT3	ATCAGCGCTCTTGCAACTCT	GGTGCACTTTGTGGAAGCTG
p-RSAD2	ATGTGGACACTGGTACCTGTCACCT	TCACCAGTCCAGCTTCAGGTCCGCC
p-IL-17	ACAAAGTCCAGGATGCCCAA	GGTGAGGTGAAGCGTTTGGA
p-GAPDH	AGCAACAGGGTGGTGGACCT	CTGGGATGGAAACTGGAAGT
h-IFN-β	GTGGCAATTGAATGGGAGGC	AGCAAAGATGTTCTGGAGCA
h-TNF-α	CAGACAGAGAGGACAGGAACCG	AGGGAGAGAGGGAGAGGAGAGT
h-RIG-I	TTCCCAGACCACAGGAATACC	GCAGGAGAACAAAGCCCAACT
h-ISG-15	CGGTGTCAGAACTGAAGAAGC	CAGACCCAGACTGGAAAGGGT
h-ISG-56	GCTTTCAAATCCCTTCCGCTAT	GCCTTGGCCCGTTCATAAT
h-IFIT3	TCAGAAGTCTAGTCACTTGGGG	ACACCTTCGCCCTTTCATTTC
h-IL-6	TGAAAGCAGCAAAGAGGC	TCAAATCTGTTCTGGAGGT
h-IL-8	TCCAAACCTTTCCACCCC	CACAACCCTCTGCACCCA
h-CXCL10	GTGGCATTCAAGGAGTACCTC	TGATGGCCTTCGATTCTGGATT
h-NF-κB	TGGACCGCTTGGGTAACTCT	CCACCAGCAGCAGCAAACAT
h-IL-17	CCAACATTTTTCTCTTCCTCA	AAAGTTCATTCTGCCCCATCA
h-RSAD2	ATGTGGGTGCTTACACCTGCTGCTT	CTACCAATCCAGCTTCAGATCAGCC
SADS-CoV N	AACGGATTCAGGGTGTGCAT	CGATTGCGAACACCAAGACC
h-GAPDH	TCTGCTCCTCCTGTTCGACAG	CCCAATACGACCAAATCCGTT
pCAGGS-flag-nsp5	GATGACGACGATAAGGAATTCGCTGG TCTTAAGAAAATGGCCC	ATTAAGATCTGCTAGCTCGAGCTGTA TGTTAACACCATACATTTGCC
pXJ-s-DCP1A	CCCGATTACGCCTCCGGATCCATGGA GTCGCTGAGTCGAGC	GCTTTAATAAGATCTGGTACCTCATA GGTTGTGGTTGTCTTTGTTC
pXJ-m-DCP1A	CCCGATTACGCCTCCGGATCCATGGA GGCGCTGAGTCGAG	GCTTTAATAAGATCTGGTACCTCATA GGTTGTGGTTGTCTTTGTTC
